# Tooth Powder

**Published:** 1876-11

**Authors:** 


Tooth Powder.—The following is an excellent recipe:

| | |
|---|---|
| White castile soap, pulv., . . . . | 4 ounces. |
| Orris-root, pulv., . . . . . | 4 " |
| Cuttle-fish bone, pulv., . . . . . | 6 " |
| Prepared chalk, pulv., . . . . | 6 " |

Flavor with sassafras or wintergreen, as desired. In the preparation of tooth-powders, great care should be taken to finely pulverize all the dry ingredients, and to reduce the hard and gritty ones to the state of impalpable powder. To insure the perfect mixture of all the ingredients, they should be stirred together until they form an apparently homogeneous powder, which should then be rubbed through a fine gauze seive.—*Ibid.*

